# Atezolizumab plus bevacizumab and chemotherapy versus bevacizumab plus chemotherapy for metastatic cervical cancer: a cost-effectiveness analysis

**DOI:** 10.3389/fphar.2024.1476256

**Published:** 2024-10-21

**Authors:** Hongfu Cai, Ling Fang, Jingwen Lin, Zhiwei Zheng

**Affiliations:** ^1^ Department of Pharmacy, Fujian Medical University Union Hospital, Fuzhou, China; ^2^ Department of Pharmacy, Cancer Hospital of Shantou University Medical College, Shantou, China

**Keywords:** partitioned survival model, cost-effectiveness analysis, atezolizumab, bevacizumab plus chemotherapy, metastatic cervical cancer

## Abstract

**Aim:**

The objective of this study is to conduct a cost-effectiveness analysis in order to evaluate the economic advantages of incorporating atezolizumab into a standard bevacizumab plus platinum regimen for the treatment of metastatic cervical cancer from the Chinese medical system perspective.

**Method:**

We developed a partitioned survival model based on data obtained from the recently published BEATcc clinical trial and economic cost data. Our model utilized a tree-based decision analysis approach to simulate two different treatment strategies for metastatic cervical cancer: the standard bevacizumab plus platinum regimen, and the addition of atezolizumab to the standard treatment regimen. The economic assessment data included the costs of the drugs, costs related to treatment-induced adverse events. The cost-effectiveness metrics used in the analysis were quality-adjusted life-year (QALY) and incremental cost-effectiveness ratio (ICER). The robustness of our model was assessed through sensitivity analysis.

**Result:**

The total costs of the atezolizumab group were $128179.56, while the costs of chemotherapy group were $42065.89. The atezolizumab group gained 3.52 QALYs, whereas the chemotherapy group gained 2.35 QALY. The atezolizumab regimen resulted in an increase of 1.17 QALYs at an incremental cost of $86113.67. This led to an ICER of $73601.43, which exceeds the willingness-to-pay (WTP) threshold of $39855.79 in China. Sensitivity analysis demonstrated none of the parameters within a margin of ±25% result in significant alterations to the analysis findings.

**Conclusion:**

Atezolizumab plus bevacizumab and chemotherapy was not to be a cost-effective option for the treatment of metastatic cervical cancer compared to bevacizumab plus chemotherapy.

## 1 Introduction

Cervical cancer is a malignant neoplasm that affects the tissues of the cervix, which is the lower part of the uterus that connects to the vagina. It is caused by persistent infection with high-risk types of human papillomavirus (HPV), primarily HPV-16 and HPV-18 (Viveros-Carreño et al., 2023). Despite the significant progress made in reducing the incidence and mortality rates of cervical cancer through the implementation of current HPV vaccinations, the global burden of this disease remains staggering (Lei et al., 2020). There continue to be approximately 6,61,021 new cases and 3,48,189 deaths attributed to this disease globally each year, positioning it as one of the top four deadliest cancers among women (Bray et al., 2024). Cervical cancer still occupies a significant position in China as the fifth most prevalent cancer among women during the year 2022, exhibiting an incidence rate of 21.18 per 1,00,000 women. Moreover, it stands as the sixth primary cause of cancer-related deaths among women, with a mortality rate of 8.06 per 1,00,000 individuals (Han et al., 2024). The management of cervical cancer primarily consists of surgical interventions, radiotherapy, and chemotherapy (Hill, 2020). In cases of recurrent or metastatic cervical cancer, the standard therapy involves a combination of platinum-based chemotherapy and bevacizumab (Enríquez-Aceves et al., 2020; Gennigens et al., 2022). Regrettably, patients suffering from recurrent or metastatic disease typically experience a poorer prognosis when treated with conventional therapeutic approaches (Watkins et al., 2023). Systemic therapy plays a pivotal role in managing patients diagnosed with metastatic cervical cancer when local interventions are not effective in controlling the disease (Li et al., 2016). Chemotherapy plays a crucial role in the treatment of metastatic cervical cancer. Platinum-based chemotherapy agents, such as cisplatin and carboplatin, are commonly used in combination with other cytotoxic drugs like paclitaxel or topotecan (Abu-Rustum et al., 2023). Despite the available treatment options, the prognosis for patients with metastatic cervical cancer remains poor (Gopu et al., 2021). The five-year survival rate for metastatic disease is significantly lower. Therefore, it is imperative to explore novel therapeutic approaches and enhance the efficacy of current treatments in order to maximize patient outcomes. Targeted therapy is a strategic therapeutic approach employed for the treatment of metastatic cervical cancer, where drugs with precise specificity are utilized to directly target specific genetic mutations or proteins found exclusively within cancer cells (Cibula et al., 2023). Bevacizumab, a monoclonal antibody that targets the vascular endothelial growth factor (VEGF), has demonstrated benefits in combination with chemotherapy for certain patients with metastatic cervical cancer (Tewari et al., 2014; Tewari et al., 2017). Recently immunotherapy has emerged as a promising approach in the treatment of metastatic cervical cancer. Immune checkpoint inhibitors (ICIs), such as pembrolizumab and nivolumab, have demonstrated clinical benefit in a subset of patients with advanced disease (Monk et al., 2023; Rodrigues et al., 2023). These agents work by enabling the immune system to recognize and eliminate cancer cells more effectively.

A recent phase 3 clinical trial named BEATcc was conducted to assess the effectiveness and safety of incorporating atezolizumab into chemotherapy, with or without bevacizumab, as a potential treatment with metastatic cervical cancer (Oaknin et al., 2024). The trial results demonstrated significant improvements in progression-free survival and overall survival when atezolizumab was combined with the standard therapy. In terms of progression-free survival (PFS), the median duration was found to be 13.7 months for the atezolizumab group, whereas it was only 10.4 months for the standard therapy group. Furthermore, at the interim overall survival (OS) analysis, the median overall survival was 32.1 months for the atezolizumab group and 22.8 months for the standard therapy group. It is worth noting that grade 3 or worse adverse events were observed in 79% of patients in the atezolizumab group, whereas 75% of patients in the standard therapy group experienced such adverse events. These findings support the consideration of atezolizumab as a new and promising first-line therapy option for metastatic cervical cancer, given its significant improvements in overall survival and progression-free survival.

While the combined administration of atezolizumab, bevacizumab, and chemotherapy has demonstrated the ability to significantly prolong the survival duration of patients afflicted with metastatic cervical cancer, it is worth noting that this particular therapeutic approach carries a greater financial burden when compared to the utilization of chemotherapy as a standalone treatment. The addition of bevacizumab to a standard chemotherapy regimen incurs an escalated cost, amounting to approximately thirteen times that of chemotherapy alone (Minion et al., 2015). Given the recent inclusion of atezolizumab, a more costly medication, in the treatment protocol, it becomes imperative to ascertain if the augmented expenses associated with this novel drug are justified by the potential health advantages, within the constraints of budgetary limitations within the healthcare sphere. In recent years, the escalating costs of pharmaceutical drugs have emerged as a grave issue encompassing governments, healthcare systems, and patients on a global scale. The exorbitant prices associated with drugs not only impose an immense burden on health budgets but also prompt the need for a comprehensive evaluation of their value and the formulation of effective healthcare policies. Consequently, the implementation of cost-benefit analysis has become a paramount tool in healthcare decision-making processes, aiming to assess the worthiness of drugs and facilitating evidence-based policy formation. Therefore, the objective of this research is to evaluate the cost-effectiveness of atezolizumab for metastatic cervical cancer from the perspective of the Chinese healthcare system.

## 2 Methods

### 2.1 Model establish

**FIGURE 1:**
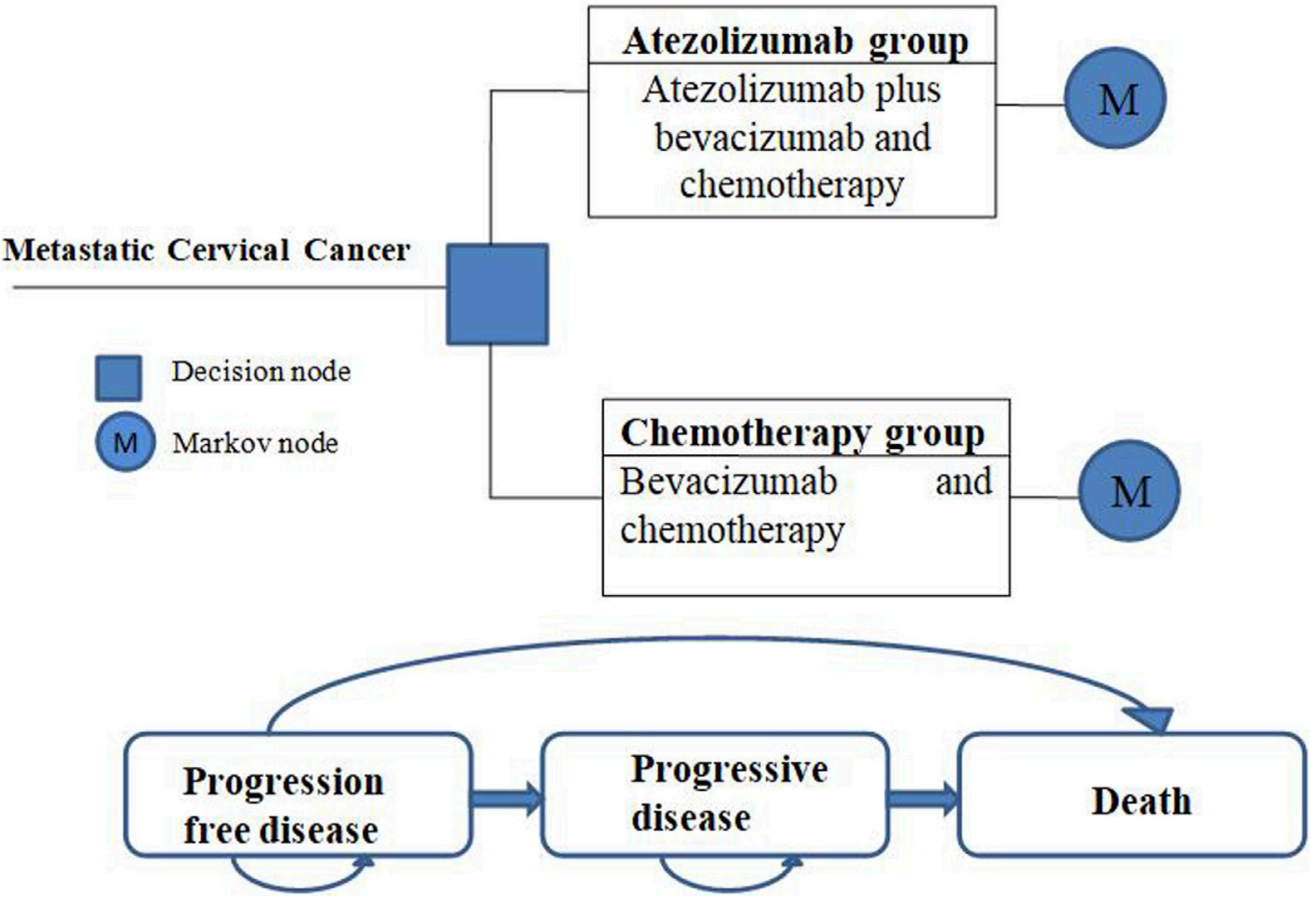
Model structure.

A partitioned survival model was constructed to simulate the progression of the disease. The model encompassed three distinct health states: progression-free disease, progressive disease, and death. These states were assumed to be mutually exclusive, meaning that a patient could only be situated in one state at any given time. The initial assumption was that all patients commenced in the PFD state, from which they could either remain in the same state or transition to another in each cycle (Figure 1).

The simulation period for the model was set at 10 years based on the fact that the overall 5-year survival rate for patients with metastatic cervical cancer is only 15% or less (Sharma et al., 2020). In the BEATcc trial, patients were administered atezolizumab or chemotherapy every 3 weeks. Accordingly, the model treatment cycle was set to match this interval of 3 weeks. To ensure the accuracy of the model, internal validation was performed by comparing the model’s predictions to the data from the BEATcc trial. Additionally, external validation was conducted by comparing the PFS and OS curves generated by the simulation with those observed in the KEYNOTE-826 clinical trial (Colombo et al., 2021). Remarkably, the simulation results closely aligned with the actual trial outcomes, further validating the reliability and validity of the model.

All costs associated with this study were converted to US dollars (USD) using the official conversion rate of Renminbi (RMB) to USD for the previous year (2023), which was determined to be 7.05 RMB per 1 USD (National Bureau of statistics of China, 2023). In accordance with the latest China Pharmacoeconomics Assessment Guidelines for the year 2020, we established a threshold for the willingness to pay (WTP) per quality-adjusted life year (QALY) at $39855.79 (Liu et al., 2020). This threshold value represents three times the gross domestic product (GDP) *per capita* in 2023 (National date, 2023). The statistical analyses and modeling for this study were performed using TreeAge Pro 2022.

### 2.2 Population and treatment

The target population for this study assumes a cohort of patients that is substantially identical to those enrolled in the BEATccl clinical trial. A total of 519 patients were evaluated to determine eligibility for inclusion in the study, of which 410 were ultimately enrolled and randomly assigned into two groups: 206 participants were allocated to the atezolizumab group, while 204 were assigned to receive chemotherapy. The mean age of the patients in the atezolizumab group was 51.0 years (with a range of 43.0–60.0 years), while the mean age of those in the chemotherapy group was 52.5 years (with a range of 43.5–61.0 years).

The clinical data for the Model treatment was derived from the randomized phase III BEATcc trial. Chemotherapy was administered intravenously and consisted of platinum (cisplatin 50 mg/m^2^ or carboplatin area under the curve of 5), paclitaxel 175 mg/m^2^, and bevacizumab 15 mg/kg every 3-week cycle. In addition, patients assigned to the atezolizumab group also received intravenous atezolizumab 1,200 mg on day 1 of each 3-week cycle. Patients who achieved a complete response after at least six cycles had the option to discontinue chemotherapy and continue with bevacizumab maintenance therapy in the chemotherapy group (and atezolizumab maintenance therapy in the atezolizumab group).

The treatment duration of the BEATcc clinical trial was utilized as the basis for our model. Specifically, in the atezolizumab group, the median duration of response was observed to be 13.6 months (10.6–21.3), while it was 8.6 months (8.0–10.6) in the group receiving chemotherapy. During the BEATcc clinical trial, two factors remained undetermined: the choice of platinum medicines, specifically cisplatin and carboplatin. Our model assumes that patients had an equal likelihood of receiving either of these medications. In order to assess their impact on economic outcomes, we will conduct a sensitivity analysis on these parameters. The occurrence of treatment-related grade 3–4 serious adverse events (SAEs) in the BEATcc clinical trial was selected based on whether they were higher than 5% in either the atezolizumab group or the chemotherapy groups.

After disease progression on the administered therapy in the BEATcc clinical trial, comparable proportions of patients in both the atezolizumab group (75 out of 138 patients, accounting for 54%) and the chemotherapy group (97 out of 166 patients, accounting for 58%) received at least one subsequent therapy. In order to conduct a comprehensive cost-effectiveness analysis, we selected a gemcitabine plus carboplatin chemotherapy regimen as the second-line treatment option for both patient groups. This decision was based on data gathered from relevant clinical trials, specifically in relation to subsequent treatment strategies adopted in cases of disease progression at the data cutoff date. Nevertheless, it is important to acknowledge the considerable uncertainty associated with choosing an optimal third-line therapy. As a result, our study assumes the implementation of a best supportive care regimen in the event of disease re-progression.

### 2.3 Model transfer probability

The Kaplan-Meier survival curve was derived from data obtained during the BEATcc clinical trial. In order to extract the Model transfer probability data, the Get Data Graph Digitizer 2.25 software was utilized. This curve was employed to estimate the probability of transitioning between three distinct health states. To estimate the distribution of the probability of survival, various statistical distributions including gamma, log-normal, log-logistic, exponential, gompertz, and weibull were evaluated. The selection of the best-fitting distribution was determined by a combination of visual inspection, the minimum value of the akaike information criterion (AIC), and the bayesian information criterion (BIC) (Ishak et al., 2013). The results are presented in Supplementary Figure S1; Supplementary Table S1. Subsequently, the log-logistic distribution was selected to simulate the PFS and OS curves for the two schemes. In order to enhance the effectiveness of our model and broaden its applicability beyond the duration of the clinical trial follow-up, a simulation approach was implemented to generate survival times based on the log-logistic distribution. Furthermore, the survival function of the log-logistic distribution over time was calculated using the formula S(t) = 1/(1 + λt^γ^) (Diaby et al., 2014), where λ represents the scale parameter and γ represents the shape parameter. The estimated values of these parameters are presented in Table 1.

**TABLE 1:** Basic input parameters to model.

Variable	Baseline value	Range		Distribution	Source
		Minimum	Maximum		
Log-logistic PFS survival model					
Atezolizumab group	γ = 1.74; λ = 0.0079	—	—	—	Oaknin et al. (2024)
Chemotherapy group	γ = 2.18; λ = 0.0052	—	—	—	Oaknin et al. (2024)
Log-logistic OS survival model					
Atezolizumab group	γ = 2.02; λ = 0.00099	—	—	—	Oaknin et al. (2024)
chemotherapy group	γ = 2.16; λ = 0.0011	—	—	—	Oaknin et al. (2024)
Atezolizumab group SAEs (grade ≥ 3) incidence					
Anaemia	0.15	—	—	Beta	Oaknin et al. (2024)
Neutropenia	0.18	—	—	Beta	Oaknin et al. (2024)
Diarrhoea	0.05	—	—	Beta	Oaknin et al. (2024)
Hypertension	0.18	—	—	Beta	Oaknin et al. (2024)
Chemotherapy group SAEs (grade ≥ 3) incidence					
Anaemia	0.08	—	—	Beta	Oaknin et al. (2024)
Neutropenia	0.25	—	—	Beta	Oaknin et al. (2024)
Diarrhoea	0.03	—	—	Beta	Oaknin et al. (2024)
Hypertension	0.16	—	—	Beta	Oaknin et al. (2024)
Drug cost (US dollar $)					
Atezolizumab per mg	3.88	2.91	4.85	Gamma	Yao (2024)
Bevacizumab per mg	1.60	1.20	2.00	Gamma	Yao (2024)
Paclitaxel per mg	0.24	0.18	0.30	Gamma	Yao (2024)
Cisplatin per mg	0.22	0.17	0.28	Gamma	Yao (2024)
Carboplatin per mg	0.086	0.06	0.11	Gamma	Yao (2024)
Costs of SAEs (grade ≥ 3) events per cycle ($)					
Anaemia	531.70	398.78	664.63	Gamma	Zheng et al. (2023)
Neutropenia	461.50	346.13	576.88	Gamma	Zheng et al. (2023)
Diarrhoea	88.38	66.29	110.48	Gamma	Chiang et al. (2021)
Hypertension	12.90	9.68	16.13	Gamma	Zheng et al. (2022)
Subsequent treatment	137.61	103.21	172.01	Gamma	Yao (2024)
Best supportive care per cycle	337.50	253.13	421.88	Gamma	Zheng et al. (2023)
Follow-up cost per cycle	55.6	41.70	69.50	Gamma	Zheng et al. (2023)
Routine laboratory examinations per cycle	92.50	69.38	115.63	Gamma	Zheng et al. (2024)
Abdominal CT per cycle	105.90	79.43	132.38	Gamma	Zheng et al. (2024)
Utility value					
Progression-free disease	0.85	0.64	1.00	Beta	Zheng et al. (2023)
Progressive disease	0.52	0.39	0.65	Beta	Zheng et al. (2023)
Anaemia	0.38	0.29	0.48	Beta	Zheng et al. (2023)
Neutropenia	0.2	0.15	0.25	Beta	Zheng et al. (2023)
Diarrhoea	0.11	0.08	0.14	Beta	Chiang et al. (2021)
Hypertension	0.1	0.08	0.13	Beta	Zheng et al. (2022)
Body surface area (m^2^)	1.64	1.23	2.05	Beta	Yue et al. (2021)
Discount rate	0.05	0.04	0.06	Beta	Yue et al. (2021)

### 2.4 Cost and utility input

The direct healthcare costs in this study are determined from the perspective of the Chinese medical system. These costs specifically include expenses associated with medication, management of adverse events, subsequent treatment, optimal supportive care, follow-up, and fees for medical testing and inspections, which is basically from the citation of published literature.

According to the report on nutrition and chronic diseases of Chinese residents (2020) and current development and practice of pharmacoeconomic evaluation guidelines for universal health coverage in China (Yue et al., 2021), the chemotherapy dose was calculated using a body surface area model, assuming a body surface area of 1.64 m^2^, body weight of 60 kg, and height of 160 cm. The dose calculation also took into account the patient’s creatinine clearance, which was estimated at 70 mL/min. To ensure accurate estimation of drug costs, we utilized the China Data Platform (https://data.yaozh.com/). This platform provided us with national median drug prices, which served as the input values for our cost model.

In this study, utility values ranging from 0 to 1 were utilized to evaluate the quality of life associated with each health condition. However, it is important to note that explicit data on utility values were not obtainable from the BEATcc clinical trial. As a result, we turned to the existing literature to source these values. Furthermore, our model acknowledges the negative impact on utility caused by adverse drug events. Detailed information on cost and utility values can be found in Table 1.

### 2.5 Sensitivity analysis

We conducted both one-way sensitivity analysis and probabilistic sensitivity analysis (PSA) to assess the stability of our model. In the one-way sensitivity analysis, we examined the impact of various parameters on the incremental cost-effectiveness ratio (ICER) by varying them within a range of ±25% of the base case value. The results of the one-way sensitivity analysis were visually represented using a tornado diagram. For the PSA we utilized a monte carlo simulation with 10,000 iterations. The parameters in the simulation were adjusted according to pre-specified distributions. Specifically, costs followed a gamma distribution, probability parameters followed a beta distribution, and utilities followed a beta distribution. The PSA results were presented using scatter plots and cost-effectiveness acceptability curves.

## 3 Results

### 3.1 Base case results

According to the analysis, the total costs incurred by the atezolizumab group amounted to $128179.56, whereas the chemotherapy group’s total costs totaled $42065.89. Over a 10-year period, the atezolizumab group experienced an increase of 3.52 QALY, while the chemotherapy group saw an increase of 2.35 QALY. Consequently, the atezolizumab regimen yielded a net gain of 1.17 QALY when compared to the chemotherapy group, albeit at an incremental cost of $86113.67. This resulted in an incremental cost-effectiveness ratio (ICER) of $73601.43, surpassing the Chinese WTP threshold of $39855.79. Based on these findings, the atezolizumab regimen cannot be deemed cost-effective within the Chinese healthcare system. Table 2 provides overview of the findings derived from this analysis.

**TABLE 2:** The results of base case results.

Base case	Atezolizumab group	Chemotherapy group
Cost ($)	128179.56	42065.89
QALY	3.52	2.35
Incremental cost ($)	86113.67	—
Incremental QALY	1.17	—
ICER ($/QALY)	73601.43	—

### 3.2 Sensitivity analysis results

**FIGURE 2:**
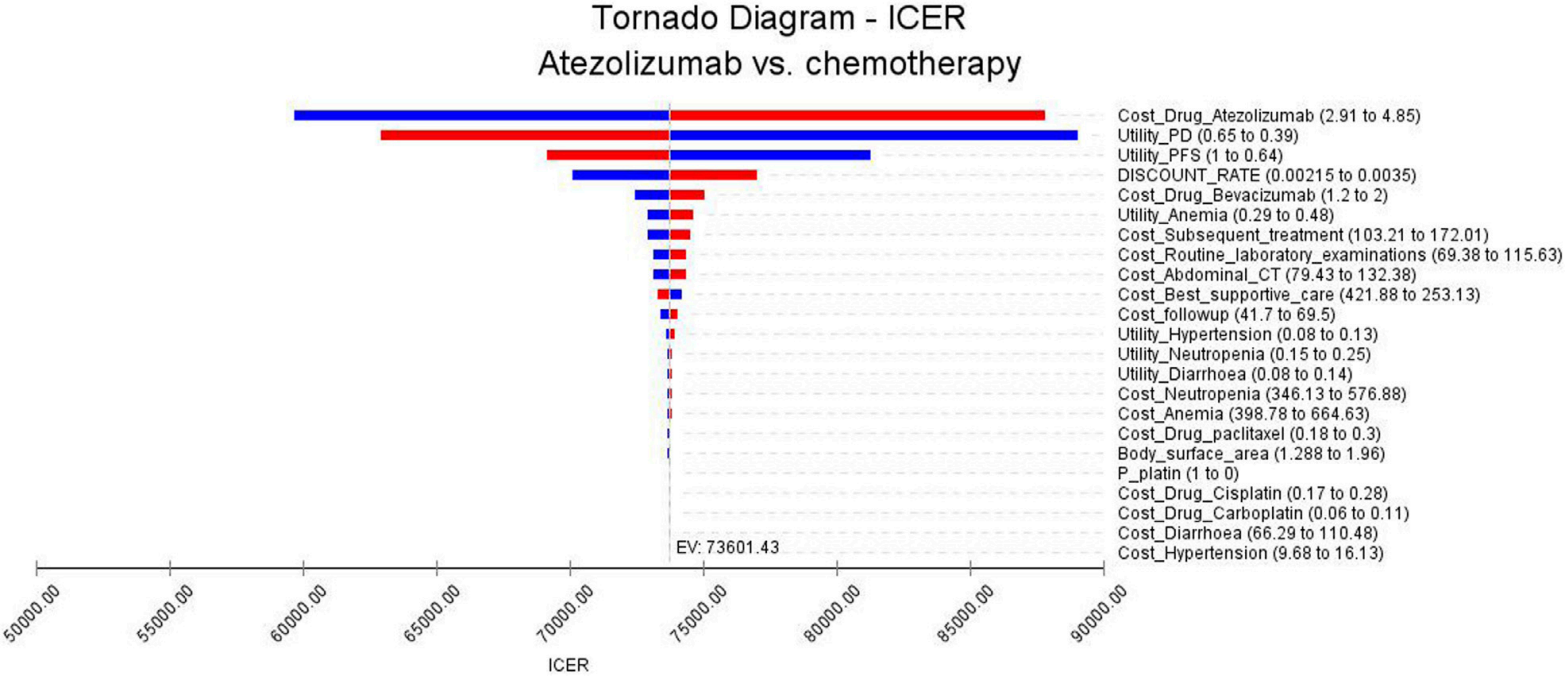
Tornado diagrams of one-way sensitivity analysis.

The findings of the one-way sensitivity analysis are presented in Figure 2. This analysis identified several key factors that significantly impacted the ICER. Notably, the cost of atezolizumab, the utility value of progressive disease, and progression-free disease were found to exert a substantial influence on the ICER. In Figure 2, the red bars depict the effect of increasing the parameter value from its base value on the ICER. Conversely, the blue bars represent the impact of decreasing the parameter value on the ICER. Notably, when all the uncertain parameters were altered, the ICER value did not fall below the threshold of WTP. It is imperative to emphasize that adjusting these parameters within a margin of ±25% did not result in significant alterations to the analysis findings. These results are consistent with the conclusions drawn from the base-case analysis.

**FIGURE 3:**
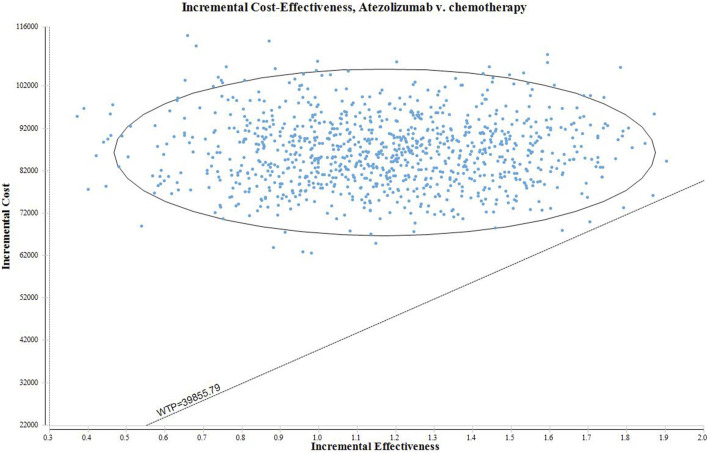
The scatter plot of PSA.

**FIGURE 4:**
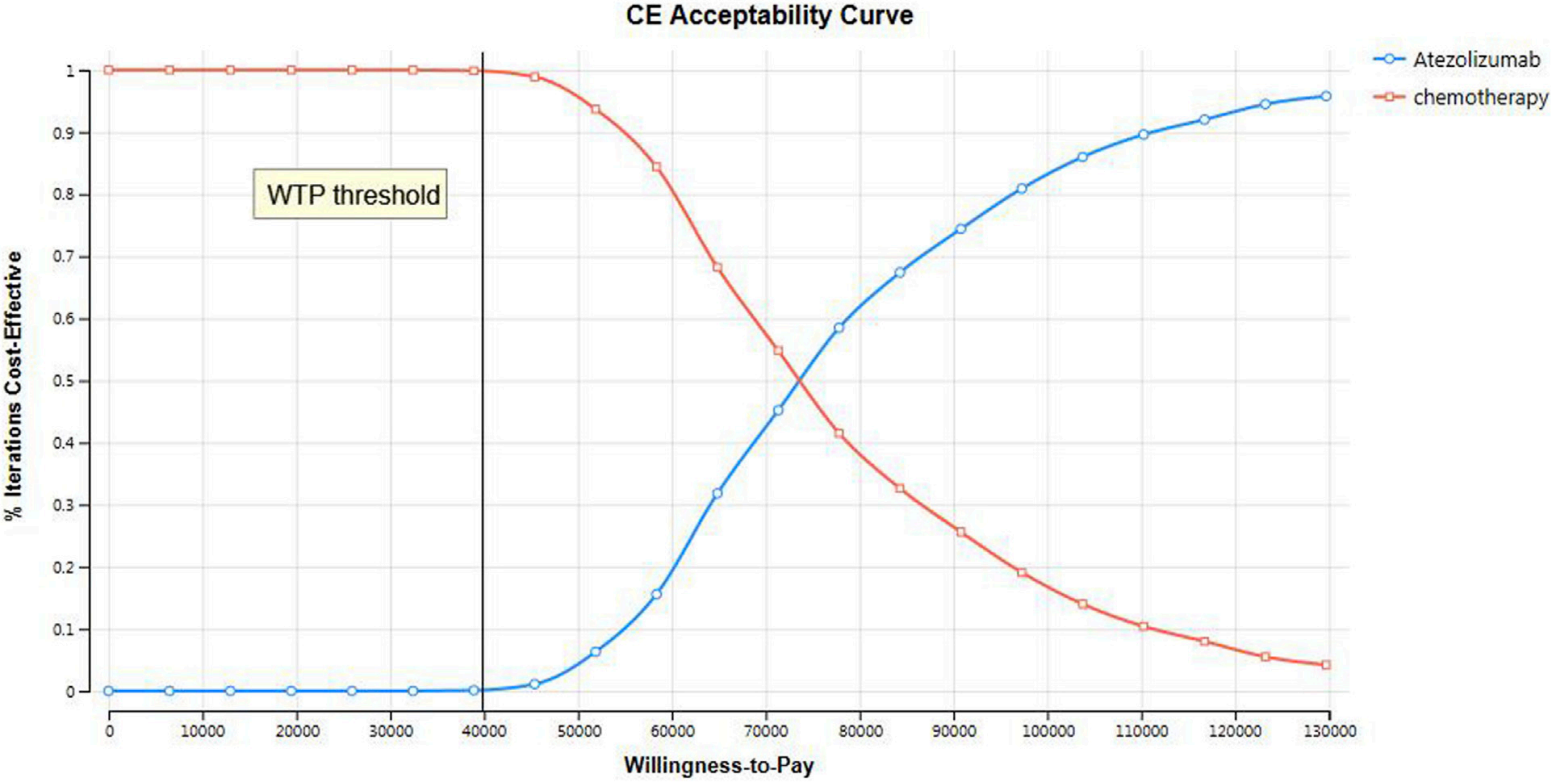
The cost-effectiveness acceptance curve of PSA.

The outcomes of the PSA are presented in Figure 3. All data points in the scatter plot exceeded the designated threshold, indicating that atezolizumab is not a cost-effective option compared to chemotherapy. The cost-effectiveness acceptance curve is illustrated in Figure 4. At a WTP threshold of $39855.79, the probability of atezolizumab having a cost-effectiveness advantage was only 0.10%. This probability increased to 54.70% when the WTP threshold was $75726.00. As the WTP threshold increased, the likelihood of atezolizumab being cost-effective rose as well. Notably, at a WTP threshold of $170726.00, the probability of atezolizumab being cost-effective reached 99.00%.

## 4 Discussion

While cervical cancer is a preventable and treatable disease, it continues to pose a significant health concern for society (Chargari et al., 2022). In comparison to other gynecologic malignancies, cervical cancer stands out due to its extensive body of robust evidence regarding prevention and screening strategies. Numerous studies have consistently demonstrated the remarkable efficacy of HPV vaccination in early adolescence, not only in preventing HPV infection but also in impeding the development of precancerous lesions and ultimately reducing the incidence of cervical cancer (Ferrari and Giannini, 2024). However, cervical cancer still occupies a significant position in China as the fifth most prevalent cancer among women during the year 2022, exhibiting an incidence rate of 21.18 per 1,00,000 women. Moreover, it stands as the sixth primary cause of cancer-related deaths among women, with a mortality rate of 8.06 per 1,00,000 individuals (Han et al., 2024). The management of cervical cancer primarily consists of surgical interventions, radiotherapy, and chemotherapy (Hill, 2020). In cases of recurrent or metastatic cervical cancer, the standard therapy involves a combination of platinum-based chemotherapy and bevacizumab (Enríquez-Aceves et al., 2020; Gennigens et al., 2022). Regrettably, patients suffering from recurrent or metastatic disease typically experience a poorer prognosis when treated with conventional therapeutic approaches (Watkins et al., 2023). Recent studies have demonstrated promising advancements in the treatment of cervical cancer, indicating that ICIs could potentially become the new standard of care for patients suffering from this condition. These inhibitors offer a novel therapeutic approach that enhances the body’s immune response against cancer cells. Moreover, the adoption of a multifaceted treatment strategy, combining agents such as carboplatin, paclitaxel, bevacizumab, pembrolizumab, and atezolizumab, as first-line therapy could significantly enhance the outcomes of patients with metastatic cervical cancer (D’Oria et al., 2024). By implementing this comprehensive treatment regimen, it is anticipated that patients would experience improved clinical outcomes and an enhanced quality of life. Notably, phase III BEATcc trials investigating the efficacy of atezolizumab, in combination with chemotherapy and bevacizumab, have demonstrated encouraging results as a first-line treatment option for patients with metastatic cervical cancer. However, the addition of atezolizumab to the treatment regimen may result in an increase in treatment costs. Therefore, it is crucial to carefully evaluate the pharmacoeconomics associated with incorporating atezolizumab therapy to determine whether the potential benefits in terms of efficacy outweigh the financial burden.

The present study aims to investigate the economic implications of adding atezolizumab therapy to the standard treatment for metastatic cervical cancer. Our study demonstrated that the addition of atezolizumab to chemotherapy yielded a modest increase of 1.17 QALYs compared to the chemotherapy alone group. However, it also resulted in a substantial incremental cost of $86113.67. The ICER was calculated to be $73601.43, which exceeded the WTP threshold in China. We conducted sensitivity analyses to evaluate the robustness of our results. However, none of the variables included in the analysis demonstrated a significant impact on the model output. This indicates that the findings of our study are consistent and not heavily influenced by specific assumptions or input parameters. Based on our analysis, it is evident that atezolizumab does not fall within the range of cost-effective treatment options when compared to chemotherapy alone, considering the cost-effective WTP threshold in China of $39855.79 per QALY.

We express our vehement disagreement with the application of cost-effectiveness analyses, specifically the conclusions presented in this study, as the singular basis for restricting the availability of atezolizumab. It is crucial that any restrictions on its usage take into account factors beyond purely economic considerations. Rather than restricting access, we propose leveraging the insights provided by these analyses to shape policy decisions and enhance the overall economic viability of atezolizumab through improvements in the healthcare insurance system (National Academies of Sciences, 2017). In recent years, the rising costs of pharmaceutical drugs have become a significant concern for governments, healthcare systems, and patients worldwide. High prices of drugs not only place a heavy burden on healthcare budgets but also limit access to life-saving treatments for patients in need. Cost-effectiveness analyses have emerged as a valuable tool to inform decision-making in healthcare systems, particularly regarding the pricing and reimbursement of pharmaceutical drugs (Hall Dykgraaf and Barnard, 2020). In the case of China, the implementation of a centralized national procurement system for pharmaceuticals has emerged as a significant and proactive response to address the financial pressures stemming from exorbitant drug prices. This strategic approach enables the Chinese government to engage in extensive negotiations with pharmaceutical manufacturers on a large scale, effectively leveraging the immense purchasing power of the nation to secure more favorable prices for crucial medications (Zhu et al., 2023). Undoubtedly, this innovative method has yielded remarkable results, as evidenced by the successful negotiations between the Chinese government and manufacturers, leading to a substantial reduction in the price of gefitinib, surpassing 50% (Lu et al., 2022). The group purchasing mechanism organized by the Chinese government has become a powerful tool for the pharmaceutical industry to reduce drug prices and increase accessibility. By integrating demand, a transparent bidding process and skillful negotiation strategies, significant price reductions have been achieved, benefiting both parties (Chen et al., 2020).

According to our one-way sensitivity analyses, we found that the price of atezolizumab significantly influenced the results of our study. A change in the price of this drug could have a substantial impact on the ICERs obtained. Notably, even with a 50% reduction in the price of atezolizumab ($45601.84/QALY), the ICERs still exceeded the WTP threshold in China. However, it is worth noting that when the price of atezolizumab was reduced to $1.47 per milligram (equivalent to 38% of the current price), the ICER ($38854.68/QALY) approached the WTP threshold ($39855.79/QALY). These findings suggest that lowering the price of atezolizumab may make this treatment regimen more financially viable and potentially improve its cost-effectiveness in China.

No cost-effectiveness analyses comparing first-line treatment with atezolizumab plus bevacizumab and chemotherapy versus bevacizumab plus chemotherapy for metastatic cervical cancer have been reported in China. Nevertheless, Lei et al. conducted a state-transition Markov model to evaluate the cost-effectiveness of bevacizumab plus atezolizumab compared to chemotherapy plus bevacizumab for the treatment of patients with persistent, recurrent, or metastatic cervical cancer (Lei et al., 2024). The analysis was performed from the perspective of healthcare payers in the United States over a 10-year time horizon. The research findings indicate that, based on the cost-effectiveness analysis, bevacizumab plus atezolizumab treatment is unlikely to be a cost-effective option when compared to chemotherapy plus bevacizumab for patients with persistent, recurrent, or metastatic cervical cancer in the United States. Furthermore, there have been several other studies conducted to examine the cost-effectiveness of ICIs in the treatment of metastatic cervical cancer. Yang Shi et al. conducted a partitioned survival model to assess the cost-effectiveness of pembrolizumab compared to placebo, using clinical data derived from the KEYNOTE-826 phase 3 randomized trial, specifically from the perspective of the United States. The study findings indicate that the incorporation of pembrolizumab into chemotherapy regimens exhibits a high cost burden and may not demonstrate cost-effectiveness for individuals with persistent, recurrent, or metastatic cervical cancer at the present pricing in the United States (Shi et al., 2022). Additionally, Gengwei Huo et al. conducted a comprehensive analysis to assess the cost-effectiveness of tisotumab vedotin as a treatment for patients with recurrent or metastatic cervical cancer in the second or third line, from the perspective of the United States payer. The study’s findings revealed that tisotumab vedotin was deemed unlikely to be cost-effective compared to traditional chemotherapy options for these patients (Huo et al., 2024).

It is crucial to acknowledge and address the limitations present within our study. Firstly, it is crucial to note that the data utilized in our research were gleaned from the clinical trials. However, in light of the ever-evolving nature of the healthcare landscape, it is imperative to emphasize the need to continuously monitor and update these findings. As novel evidence emerges and both costs and efficacy fluctuate within the field, it becomes increasingly pertinent to stay abreast of these developments to ensure the ongoing accuracy and relevance of our research outcomes. Secondly, there is a need to conduct comprehensive investigations to assess the long-term effectiveness and cost-effectiveness of the intervention across various patient subpopulations. This could involve examining the sustained benefits and outcomes of the intervention beyond short-term studies, while considering factors such as age, socioeconomic status, and comorbidities. It is important to understand how the intervention may differ in its impact and cost-effectiveness among diverse patient groups, as this can inform personalized and evidence-based healthcare decisions. Thirdly, it is important to acknowledge that our study assumes a specific cost for second-line therapy following disease progression. However, in practice, the selection of subsequent treatment regimens may vary based on the individual circumstances of each patient. Despite this potential variation, we find solace in the results of our thorough one-way sensitivity analyses, which consistently demonstrate that the ICER remain above the threshold of WTP, even when altering the estimated range of subsequent treatments. This robustness of our findings reinforces the validity and reliability of our study. Lastly, a limitation of our study lies in the exclusion of grade 1 or 2 adverse events from the analysis, considering their minimal impact on clinical outcomes. While this approach facilitates concise modeling, it may not fully reflect the overall impact of treatment-related toxicity on patient prognosis. However, it is encouraging that our sensitivity analyses indicate that even grade 3 or higher adverse events fall within the range of variation and do not alter our conclusions.

## 5 Conclusion

The incorporation of atezolizumab into combination chemotherapy regimens is accompanied by exorbitant costs and may not be cost-effective for metastatic cervical cancer in China. Nevertheless, it is suggested that by reducing the price of atezolizumab by an additional 38% to $1,764 per 1,200 mg, the treatment has the potential to attain cost-effectiveness when gauged against the prevailing WTP threshold in China.

## Data Availability

The original contributions presented in the study are included in the article/Supplementary Material, further inquiries can be directed to the corresponding author.

## Supplementary material

SUPPLEMENTARY FIGURE S1
**(A)** Modes simulation visual progression-free survival curve of atezolizumab group; **(B)** Modes simulation visual progression-free survival curve of chemotherapy group; **(C)** Modes simulation visual overall survival curve of atezolizumab group; **(D)** Modes simulation visual overall survival curve of chemotherapy group.
