# Supplementary material for: Atezolizumab plus bevacizumab and chemotherapy versus bevacizumab plus chemotherapy for metastatic cervical cancer: a cost-effectiveness analysis
Source: Front Pharmacol. 2024 Oct 21;15:1476256. doi: 10.3389/fphar.2024.1476256 (PMC11532157; doi:10.3389/fphar.2024.1476256)
Supplement: Supplementary file 1 [file Table1.DOCX]

**Supplementary Table 1. Comparison of survival models distribution**

|  | AIC | | BIC | |
| --- | --- | --- | --- | --- |
|  | Atezolizumab group | Chemotherap**y** group | Atezolizumab group | Chemotherap**y** group |
| PFS |  |  |  |  |
| Weibull | 1167.63 | 1218.01 | 1174.28 | 1224.65 |
| **Log-logistic** | **1135.51** | **1187.61** | **1142.17** | **1194.24** |
| Log-normal | 1141.28 | 1192.45 | 1147.94 | 1199.09 |
| Gompertz | 1174.18 | 1238.23 | 1180.84 | 1244.86 |
| Exponential | 1172.52 | 1239.52 | 1175.85 | 1242.83 |
| Gamma | 1161.41 | 1207.60 | 1168.07 | 1214.5 |
| OS |  |  |  |  |
| Weibull | 968.29 | 1108.21 | 974.95 | 1114.85 |
| **Log-logistic** | **963.70** | **1104.61** | **971.45** | **1111.25** |
| Log-normal | 964.79 | 1104.75 | 970.36 | 1111.38 |
| Gompertz | 980.18 | 1123.18 | 986.84 | 1129.82 |
| Exponential | 995.02 | 1144.59 | 998.35 | 1147.91 |
| Gamma | 965.59 | 1104.92 | 972.24 | 1111.56 |

AIC: Akaike information criterion; BIC: Bayesian Information Criterion; OS: Overall survival; PFS: Progression-free survival;
