# Supplementary figures and images for: Atezolizumab plus bevacizumab and chemotherapy versus bevacizumab plus chemotherapy for metastatic cervical cancer: a cost-effectiveness analysis
Source: Front Pharmacol. 2024 Oct 21;15:1476256. doi: 10.3389/fphar.2024.1476256 (PMC11532157; doi:10.3389/fphar.2024.1476256)

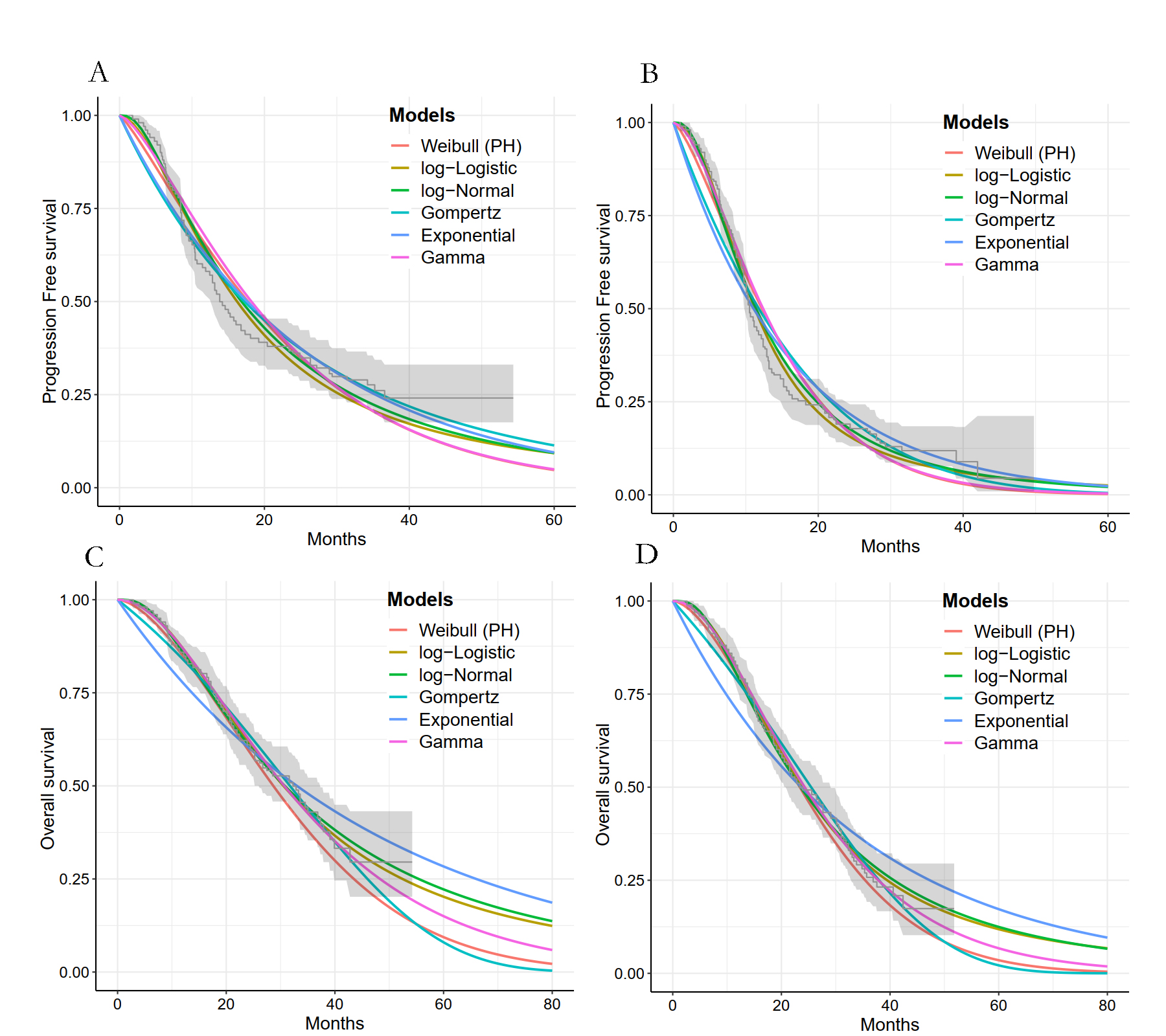

Supplement: Supplementary file 2 [file Image1.JPEG]
